# Multiple Sclerosis—A Demyelinating Disorder and Its Dental Considerations—A Literature Review with Own Case Report

**DOI:** 10.3390/brainsci13071009

**Published:** 2023-06-29

**Authors:** Khalid Al Johani, Mashael Fudah, Mohammad Al-Zahrani, Hassan Abed, Kumar Chandan Srivastava, Deepti Shrivastava, Marco Cicciù, Giuseppe Minervini

**Affiliations:** 1 Department of Oral Diagnostic Sciences, Faculty of Dentistry, King Abdulaziz University, Jeddah 21589, Saudi Arabia; 2 Department of Periodontics, University Dental Hospital, King Abdulaziz University, Jeddah 21589, Saudi Arabia; 3 Department of Periodontics, Faculty of Dentistry, King Abdulaziz University, Jeddah 21589, Saudi Arabia; msalzahrani@kau.edu.sa; 4 Department of Basic and Clinical Oral Sciences, Faculty of Dentistry, Umm Al-Qura University, Makkah 24382, Saudi Arabia; hhabed@uqu.edu.sa; 5 Department of Oral and Maxillofacial Surgery & Diagnostic Sciences, College of Dentistry, Jouf University, Sakaka 72345, Saudi Arabia; 6 Periodontics Division, Department of Preventive Dental Sciences, College of Dentistry, Jouf University, Sakaka 72345, Saudi Arabia; sdeepti20@gmail.com; 7 Department of Periodontics, Saveetha Dental College and Hospitals, Saveetha Institute of Medical and Technical Sciences, Saveetha University, Chennai 602105, India; 8 Department of Biomedical and Surgical and Biomedical Sciences, Catania University, 95123 Catania, Italy; mcicciu@unime.it; 9 Multidisciplinary Department of Medical-Surgical and Dental Specialties, University of Campania Luigi Vanvitelli, 80138 Naples, Italy

**Keywords:** multiple sclerosis, oral medicine, oral manifestations, trigeminal neuralgia, demyelinating diseases, biological products

## Abstract

Multiple sclerosis (MS) is a chronic, autoimmune condition that primarily affects the myelin sheath covering the neurons of the central nervous system, including those of the brain and spinal cord. Although the etiology is not completely understood, various factors, such as genetic infections and environmental background, play a role in the pathogenesis. Repeated active episodes of MS characterized with marked inflammation results in the scarring of particular nerve segments, and eventually results in functional impairment over a period of time. Based on the clinical course of the disease, four clinical types of MS have been identified, with the relapsing–remitting type being the commonest. MS is known to occur more commonly in females in the age group of 20–40 years. Dysarthria, fatigue, muscle spasm, and numbness are the common presenting symptoms of MS. Diagnosis is generally achieved with MRI brain scans, showing demyelination plaques and lumbar puncture. Treatment of MS’s acute phase includes high doses of corticosteroids; whereas preventive treatment of MS includes the prescription of immunosuppressive therapy, including biologics. A large group of MS patients present with oral manifestations, including dysphagia, dysarthria, temporomandibular joint (TMJ) disturbances, facial palsy, and chronic periodontal diseases. Other typical oral manifestations seen in MS patients include trigeminal neuralgia, paresthesia, or orofacial pain. Dental treatment and following drug prescription needs to be tailored to each patient, as there is a possibility of drug interactions. This paper presents a comprehensive, updated review of MS, with emphasis on oral manifestations and dental considerations. Additionally, it presents a case of a 40-year-old female diagnosed with MS that was presented to a dental hospital. The report discusses the oral manifestations and dental management.

## 1. Introduction

Multiple sclerosis (MS) is a chronic autoimmune condition where autoantibodies cause the destruction of the myelin sheath covering the nerves of the central nervous system [1]. MS is a highly varied entity, and the course of the disease is unpredictable. Initially, in most of the patients, it shows signs of reversible neurological deficit. However, once the condition becomes chronic, it undergo progressive neurological deterioration [1,2]. The etiology of MS is not clearly understood. However, there are a few environmental factors, such as infection with the Epstein virus, human herpesvirus 6, and low levels of vitamin D, that are thought to be associated with MS [3]. It is characterized by sensory, motor, and cognitive disorders manifested as numbness, ataxia, muscle fatigue, and muscle spasticity [3]. There are four major types of MS, with relapsing–remitting (RRMS) being the predominant [4]. The role of the dental clinician includes the identification of an MS patient, delivery of appropriate dental treatment, and timely referral for medical intervention. The clinical phase of MS, level of disease control, current prescriptions, degree of motor impairment, and level of fatigue dictates the nature and duration of dental treatment. Additionally, drug therapy advised after the dental treatment needs to be conducted cautiously in order to prevent drug interactions.

This paper attempts to review updated etiology, pathogenesis, clinical features, diagnosis, and management of MS. However, the paper primarily focuses on orofacial presentation and the dental management of these patients. Additionally, a brief systematic review of published reports discussing orofacial manifestations is presented. Lastly, oral manifestations and dental management is discussed through a case report of a 40-year-old female MS patient.

## 2. Multiple Sclerosis (MS)

MS is a chronic demyelinating disorder that primarily affects the myeline covering of neurons of the central nervous system. The following section discusses the various aspects of MS.

### 2.1. Etiology

MS is an autoimmune, cell-mediated disorder. Although the particular etiological agents are not specified, there are a few triggering factors that are proposed to play a role in the etiopathogenesis of MS. These factors can be grouped into environmental and genetic [5]. The earlier factor primarily involves viral infections from the Epstein–Barr virus (EBV) and human herpesvirus 6 (HHV-6). Although the exact mechanism is not understood, individuals with low levels of vitamin D have reported high incidences of MS. This could be a possible explanation of the higher occurrence of MS in geographical areas that are far from the equator, and thus receive less sunlight, the natural source of vitamin D [6].

According to another theory, genetic mutation in a particular susceptibility gene, known as HLA DR-2, is responsible for the development of MS. As a result of this mutation, the antigen-presenting cells (APC), such as dendritic cells or macrophages, exhibit an exaggerated immune response when they encounter antigens from any of the above-mentioned environmental factors [7].

### 2.2. Pathogenesis

MS is a type IV hypersensitivity reaction (HSR), also known as cell-mediated HSR, which is characterized by the destruction of myelin and its related cells. The antigen of unknown nature (possibly environmental factors such as EBV and HHV-6), when entering the body, is phagocytosed by the antigen-presenting cells (APC), mostly, the macrophages. Later, they present the antigen to the helper T-cells (CD4+), which identify the antigen as a mimic of myelin protein, and hence, the CD4+ cells become primed. The CNS is separated from the vascular bed through a blood–brain barrier (BBB), which is permeable to very specific entities. The primed CD4+ cells bear a specific ligand/surface molecule that allows them to cross the BBB and reach to the neurons present in the brain [8].

The oligodendrocytes are responsible for producing a protective covering, known as the myelin sheath, that covers the axons of neurons present in the central nervous system (CNS). The primed CD4+ cells identify certain proteins of the myelin sheath and start adhering to it so as to get activated. Later, upon activation, the T-cells release pro-inflammatory cytokines, including interleukin 1 (IL-1), interleukin 6 (IL-6), and tumor necrosis factor alpha (TNF-α). These cytokines bring about various changes that further intensify the inflammatory response. The changes include vasodilation, alteration in the capillary permeability, and increased expression of adhesion markers of the endothelial layer to facilitate entry of white blood cells (WBCs) [9]. Additionally, the cytokines are leached into the blood stream, and attract B lymphocytes through chemotactic response. Another cytokine, known as interferon gamma (INF-γ), plays an important role in attracting macrophages to the site of inflammation. Later, the B lymphocytes convert into plasma cells and start producing the antibodies that are targeted towards myelin proteins (autoantibodies). In addition, the macrophages start engulfing the oligodendrocytes. With the destruction of the myelin sheath and its cells, the damaged nerve leaves behind a plaque or sclera. With damage occurring at multiple sites, resulting in plaques at the site of destruction, the condition resulting from such damage is identified as “multiple sclerosis” [10].

Typically, in MS, the disease undergoes a cyclic pattern, with episodes of demyelination followed by repair initiated by regulatory T-cells [4]. These specific T-cells release another group of chemicals known as anti-inflammatory cytokines, such as interleukin 10 (IL-10) and transforming growth factor beta (TGF-β). They slow down the inflammatory process and initiate the repair of oligodendrocytes. In the early phases of MS, the cells undergo considerable repair and formation of new myelin sheaths, known as remyelination. However, with the progression of the disease, the process of remyelination slows down, and the damage becomes irreversible with the loss of axons [11].

### 2.3. Epidemiology

In the year 2020, a global survey was conducted where the prevalence data of MS was gathered from 81 counties. The findings were compared against a similar global survey conducted in the year 2013. The findings showed an increase in the population suffering from MS, reaching to 2.8 million, with a global prevalence of 35.9/100,000 people [95% CI: 35.87, 35.95]. Of the 81 countries that participated in the study, only 14% of countries depicted a stable or declining number of MS cases since the 2013 survey. Additionally, a striking observation was reported, where 47 countries documented a rise in the pediatric population (less than 18 years old), with more than 30,000 cases of MS [12].

The prevalence is rising in the Arabian Gulf at a rate of 2.3% per year since 1986 [13,14,15]. In Saudi Arabia, MS is affecting about 40.4 persons per 100,000 in the general population, and 61.95 per 100,000 Saudi nationals [16]. Mohammed AlJumah et al. [17] studied the secondary data of 2516 patients gathered from the national registry of Saudi Arabia pertaining to MS. They found about 12.8% of all registered patients revealed a contributory family history (FH-MS). Furthermore, among the group of FH-MS patients, 56.3% of them revealed parental consanguinity, and 42.53% reported having siblings suffering from MS [17].

In a recent paper, Bunyan and coworkers (2021) [18], utilizing the Saudi National Registry, estimated the prevalence of pediatric MS to be 2.73/100,000 of the Saudi pediatric population, and the overall projected prevalence was estimated to be 14.33/100,000 of the Saudi pediatric population. There were 287 MS pediatric patients in the register with a median age at diagnosis of 16.0 years, and girls were more prevalent than boys (female 74.2%; male 25.8%) [18].

MS has been reported to run in families, and this fact supports a genetic background for this disorder [19].

### 2.4. Clinical Types

MS has four major clinical types, namely, relapsing–remitting (RRMS), secondary progressive (SPMS), primary progressive (PPMS), and progressive–relapsing (PRMS), where the 90% of cases are classified as RRMS. The latter is characterized as having multiple episodes of disease flare, followed by a brief repair, but the degree of nerve deficit will increase with each episode. On the other hand, SPMS shows a similar pattern of flare and repair as seen in RRMS in the initial phase, but later acquires constant steady exponential demyelination, resulting in major neurological deficit and disability. The remaining two clinical types are rare, but are considered more severe forms. In PPMS, there is constant steady demyelination without any remyelination, whereas in PRMS, there is a combination of steady progressive demyelination and intermittent flare episodes, which elevates the level of neurological deficit and damage [20].

### 2.5. Clinical Features

The symptoms of MS are highly variable, as they largely depend on the location of the plaques. The disease is generally seen to occur in the age group of 20–40 years, and more commonly affecting females. The disease generally follows a course of bouts, which usually last for weeks to months, followed by a period of remission [21].

The clinical course varies widely between patients. In addition, the signs and symptoms of the disease also vary, and they depend on the demyelinated areas of the central nervous system. The symptoms of MS usually manifest as Charcot’s neurologic triad. This is characterized as dysarthria, nystagmus, and intention tremors. Plaques in the brain stem affect the motor innervation to the oropharyngeal muscles, leading to compromised movements of these muscles, including speech, mastication, and swallowing [22]. Plaques affecting the motor and sensory cranial nerves innervating the eyeball affects the vision and ocular movements. Patients might complain of complete loss of vison, blurred vision, a dark spot in the center of the field of vision, or double vision [23]. When plaques are seen in the motor tract of the spinal cord, it results in muscle weakness, spasms, tremors, ataxia, or even paralysis. When demyelination plaques are seen in sensory pathways of the spinal cord, it presents as paresthesia and a numbness or tingling sensation of the skin. Lastly, plaques might occur in the autonomic nervous system, where patients will complain of constipation and urinary incontinence. Furthermore, cognitive disability, such as poor concentration, depression, and anxiety, are also reported in various patients [24].

### 2.6. Diagnosis

MS is usually diagnosed clinically. A detailed history and clinical examination, including neurological examination, is important for the diagnosis and estimation of the extension of diseases [1]. Cerebrospinal fluid (CSF) analysis and magnetic resonance imaging (MRI) are the main factors/criteria for the diagnosis of MS; however, a long list of laboratory tests are included in the diagnostic process to exclude other disorders that mimic MS clinically [25].

Among the different advanced imaging modalities available for soft tissue imaging [26], MRI of the brain and spinal cord with and without contrast is considered as the first line of the diagnosis. The typical areas to be scanned for demyelination plaques include the periventricular matter, brain stem, spinal cord, and cerebellum. The appearance of multiple hyperintense lesions (also known as Dawson’s fingers) on sagittal/axial planes of T2-weighted sequences are of diagnostic significance. With the use of contrast, a hyperintense lesion on a T1 sagittal sequence indicates an acute lesion [27].

The visual evoked potential (VEP) test is considered as the second line of the diagnostic approach. The reduced action potential of the optic nerve is recorded in a graphical manner after a specific stimulus is introduced to the eye. The decreased conduction velocity of the optic nerve is a result of demyelination of the oligodendrocytes [28].

Lastly, lumber puncture is an adjunctive test for MS, where the CSF is withdrawn and examined for elevated levels of specific IgG antibodies. These antibodies reveal “oligoclonal bands” upon electrophoresis. Additionally, CSF can also display “pleiocytosis”, where there is a marked increase in WBCs, particularly plasma cells and macrophages [29].

### 2.7. Differential Diagnosis

Patients presenting with an attack of ill-defined pain and hyperintense lesions on T2-weighted MRI images should be investigated in a broader perspective. Various conditions of different etiology (including inflammatory, such as transverse myelitis; infectious, such as multiple metabolic; immunological, including sarcoidosis; and spinal cord neoplasms) should be considered in the differential diagnosis [30]. Careful questioning, detailed evaluation of MRI scans, and additional investigation, including gadolinium enhancement of the pia and white matter, should be carried out to exclude other conditions [31].

### 2.8. Medical Management

There is no specific treatment for MS patients. However, patients may receive corticosteroids (prednisone, methylprednisolone), monoclonal antibodies, azathioprine, methotrexate, and cyclophosphamide [5].

MS adversely affects the quality of life of affected patients and their families. Also, it presents a challenge to the attending clinicians medically and psychosocially [32].

Different agents have been recommended for use in the acute phase of the disease, including intravenous (IV) methylprednisolone, ACTH, corticosteroids, and plasmapheresis. Intravenous immunoglobulin (IVIG) (2 g/kg over 3–5 days) may be used in patients who are contraindicated for IV corticosteroids and plasmapheresis [25].

A wide variety of agents may be employed in the management of MS, including fingolimod, siponimod, natalizumab, ocrelizumab, cladribine, and alemtuzumab. A detailed algorithm for the management of patients with MS can be found in Yamout et al., 2019 [25].

The treatment of MS primarily involves two approaches. The first approach is to manage and treat the acute exacerbation of the disease, which can be characterized by decreased visual deficit, extreme weakness, or paralysis. During such acute or relapsing episodes of the disease, the treatment includes intravenous administration of high doses of corticosteroids, such as methyl prednisolone or prednisolone. The corticosteroids have a potent anti-inflammatory property, which is exploited here for the benefit of the patient. They block the T-cell release of cytokines, including IL-1, IL-6, TNF-α, and INF-γ, and thus minimize their effects on the vascular system, such as vasodilation, capillary permeability, and chemotaxis [33]. Another treatment modality considered in acute situations is plasmapheresis, where the plasma is made to flow through channels and filters, thus flushing away the pathogens and infusing fresh plasma [34].

Supportive treatment is also administrated at the same time, which includes antispasmodic agents, such as baclofen. Additionally, a supportive treatment regime includes the management of paresthesia by prescribing drugs such as gabapentine and pregabaline. Antidepressant drugs should also be considered for the management of depression [35].

The second arm of treatment for MS includes preventive management, where the emphasis is laid on reducing the symptoms and frequency of relapsing episodes, and minimizing the defect. These disease-modifying therapies involve immunosuppression drugs. This category includes interferon β, which acts by inhibiting the T-helper cells in order to block the release of cytokines [36]. Another dug in this category is glatiramer acetate, which also inhibits the T-cells and subsequently suppresses the release of the cytokines responsible for triggering the inflammation. Other popular drugs include ocrelizumab and natalizumab. The former inhibits the activity of B-cells and plasma cells, thus decreasing the production of autoantibodies. The latter acts by inhibiting the B and T lymphocytes the from invading/crossing the blood–brain barrier, so that a major inflammatory response at the site of lesion can be prevented [37].

## 3. Dental Considerations

With the recent advances in the management of various chronic disorders, including MS, more cases of MS have been seen in dental offices. Dentists play an important role in the management of this group of patients in many aspects, such as identifying the undiagnosed condition and improving oral complications, including oral hygiene, and thus aim to improve the quality of life (QoL) [38].

### 3.1. Role of Dentist in Multiple Sclerosis

The role of a dentist in the management of MS starts with the identification of MS in an undiagnosed case. The identification is generally based on history and clinical examination. Typical presentation includes a facial pain of unknown origin, tingling or numbness of the extremities, disturbances in vision, and muscle weakness. The above symptoms seen in a young female with a progressive nature of symptoms forms the ground for further investigation into MS. Hence, proper identification and later referral to a specialist for confirmation of diagnosis and management is the primary role of a dentist [39].

### 3.2. Dental Treatment Considerations

Maintaining oral health and providing dental treatment poses a challenge in patients with MS. Firstly, the daily oral hygiene is compromised due to muscle weakness and pain. In addition to that, long periods of fatigue and mobility impairment imposes a barrier for MS patients to travel to receive dental treatment. Also, a lack of knowledge among dentists to manage this group of patients contributes to the situation [40].

Delivery of dental treatment depends primarily on three factors, namely, current phase of disease, degree of motor impairment, and level of fatigue. Elective dental treatment is not advisable during the relapse phase of the disease, whereas the ideal time to provide treatment is during the remission phase. The emergency treatment must be performed with caution as the medications prescribed for MS can have dental implications [41]. Anticholinergic and tricyclic antidepressants cause dryness of mouth, resulting in a burning sensation. In extreme cases, salivary substitutes or pilocarpine can be prescribed for relief [42].

Patients with stable disease symptoms and reduced muscle spasm can be good candidates for dental treatment. However, patients exhibiting advance stages of MS with pronounced muscle spasm pose difficulties, including the need of support while sitting and rising from the dental chair, being non-ideal subjects for prosthetic and reconstructive treatment, and difficulty in maintaining daily oral hygiene [43]. MS patients typically experience maximum fatigue in the afternoon, so it is advisable to arrange short morning appointments.

The use of local anesthesia with a vasoconstrictor does not impose any additional threat; however, the use of nitrous oxide is controversial, as it may cause demyelination [44]. Generally, MS patients might be undergoing long-term corticosteroid therapy, hence alteration/supplementation of corticosteroids should be considered before stressful dental procedures. Implementation of stress reduction protocol will be beneficial for MS patients [45].

According to the literature, periodontal diseases are more prevalent in MS patients, and in many cases, this might be the presenting complaint for the dentist. Gingival inflammation and eventual progression of periodontal diseases will compromise the quality of life. Timely assessment of periodontal health through clinical and biochemical means and intervention is of utmost importance. Depending on the severity of the case and medical status of the patient, the treatment plan should be tailored for every patient. To begin with non-surgical periodontal therapy along with a prescription of herbal or CHX mouthwash is practiced [46,47,48]. Only after the resolution of the disease and inflammation is the sub-gingival therapy instituted. A strict follow-up is key to successful management.

### 3.3. Oral Manifestations

The first signs and symptoms of MS may be initially seen in the orofacial area, including dysarthria, Lhermitte’s sign (electrical sensation down the spine on neck flexion), visual disturbances, facial numbness or pain, and facial palsy or spasm. Additionally, trigeminal neuralgia, glossopharyngeal neuralgia, neuropathy, burning, tingling, and reduced sensation in the affected regions are also seen as initial or presenting complaints [42].

In a large sample of MS patients (500 patients), 88.6% of the patients had orofacial clinical manifestations that included visual disorders (80.4%), TMJ disorders (58.2%), dysarthria (42.1%), dysphagia (26.6%), facial palsy (19%), and trigeminal neuralgia (7.9%) [41,49]. A brief list of studies that reported oral manifestations are mentioned in Table 1.

There are several oral manifestations seen in MS, which can be either as a result of the disease or can be due to the effect of medications taken for the treatment of MS. The most common oral symptoms include trigeminal neuralgia (TN), oral and perioral paresthesia, and dysarthria. TN is seen to be 400 times more likely to occur in MS patients, and is managed with carbamazepine, clonazepam, gabapentin, or a surgical approach in recalcitrant cases [50].

According to a few reported cases, TMJ disorders are also seen in MS, in addition to other types of orofacial pain, such as TN. Patients with TMJ disorder generally present with pain originating around the TMJ, such as in the pre- or post-auricle area, and is transferred onto the orofacial and neck region, including temporal, occipital, malar, and cervical regions. The pain can involve either side or bilateral with varying severity [51,52,53,54,55,56]. Dysarthria is another common manifestation in MS. It includes scanning type of speech and difficulty while eating, swallowing, and speech [57,58].

Other oral manifestations include periodontal disease, including gingival inflammation. Furthermore, increased prevalence of dental caries, possibly due to xerostomia, are also prevalent oral findings among this group of patients [39]. Additionally, inability to maintain oral hygiene might also be the possible reason for higher prevalence of dental caries and periodontal diseases.

MS patients also exhibit xerostomia due to the disease process and prescribed medications, including tricyclic antidepressants, anticonvulsants, and proton pump inhibitors. This leads to high caries rates, which become evident with high DMFT, halitosis, and functional difficulties [49].

## 4. Database Search and Review of Literature

In order to further explore the pattern and prevalence of oral manifestations of MS, an attempt was made to perform an extensive search of different databases. Three primary databases, namely, PubMed, Scopus, and Cochrane, were selected. Initially, the concepts were identified to generate the search strategy. “Multiple sclerosis” and “oral manifestations” were the two main concepts that were used to conduct the search. Other keywords and MeSH terminology were incorporated and added using Boolean, truncation, and quotes to formulate the final search strategy (Table 1).

**Figure 1:**
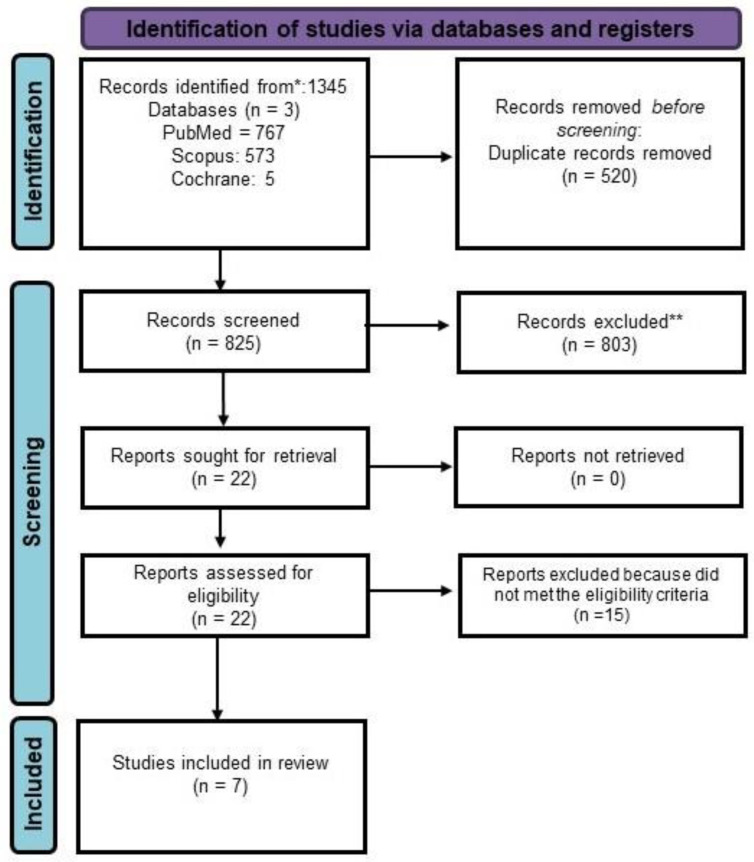
PRISMA Flowchart. * Consider, if feasible to do so, reporting the number of records identified from each database or register searched (rather than the total number across all databases/registers). ** If automation tools were used, indicate how many records were excluded by a human and how many were excluded by automation tools.

Later, the search was performed on the databases and, according to the recent PRISMA guidelines, the articles were screened. While conducting the search, filters such as “English language”, “type of study”, and “species–human” were selected. The search was not confined to any time frame, rather, the search was performed for an extended time frame. The articles were screened based on the pre-defined inclusion and exclusion criteria. Cross-sectional and clinical studies on MS with emphasis on oral health care or oral manifestations were included in the search. However, case reports, literature reviews, systematic reviews, and meta-analyses were excluded. According to the PRISMA guidelines, the identification, screening, and choosing of the studies was performed. A total of 1345 studies were initially found from the three databases: PubMed (n = 767), Scopus (n = 573), and Cochrane (n = 5). In total, 520 duplicate records were removed, and a total of 825 articles were screened. After going through the titles and abstracts, the articles were screened, and 22 articles were sourced for detailed study. Based on the eligibility criteria, the articles were further sorted, and eventually, seven studies were included for the current review (Figure 1). The key features of the seven studies are summarized in Table 2.

## 5. Management of Oral and Craniofacial Manifestations

Different agents were used in the management of neuroglia, including carbamazepine, lamotrigine, gabapentin, topiramate, phenytoin, baclofen misoprostol, NSAIDS, and intravenous corticosteroids, either alone or in combination, were found to be affective in most of the cases, especially in the early stages of the disease [64]. Also, a few surgical procedures, such as percutaneous procedures, gamma knife radiosurgery, microvascular decompression, and percutaneous retrogasserian glycerol rhizotomy, may be employed [57].

Muscle spasm of the face and pharynx was managed effectively by using antiepileptic agents, lidocaine, botulinum toxins, or cannabinoids.

Burning dysesthesias are treated with tricyclic antidepressants (amitriptyline) and antiepileptics (gabapentin) [49].

The emphasis in the management plan should be focused toward prevention and regular checkup. Oral hygiene instructions, mouthwashes, and regular dental visits should to be included in the maintenance program. Fluoride application and other preventive measurements have to be adapted [51].

Dentists have to be knowledgeable about the medications used in the management of MS, their side effects, and drug interactions with other medications prescribed by the dentist (Table 3).

Patients with MS receive multiple drugs, and this may interfere with the medications prescribed by the dentist, or may have a direct effect on their oral health. Also, some medications need special precautions, such as monoclonal ABs and corticosteroids [43].

MS may result in trigeminal neuralgia, a painful condition affecting the orofacial area. The dentist may play an important role in the diagnosis or the management of this condition.

## 6. Case Report

**Figure 2:**
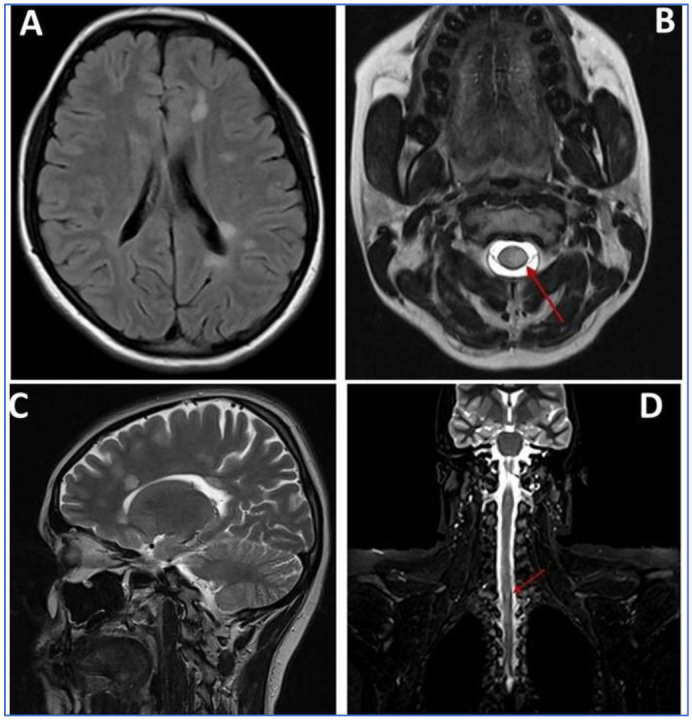
MRI scan images (**A**–**D**): (**A**) axial T2-weighted FLAIR MRI through the brain at the level of the ventricles showing multiple well-defined foci of increased signal intensity seen adjacent to left posterior aspect of periventricular white matter; (**B**) fat-suppressed axial T2-weighted MRI showing lesion around the spinal cord (Red Arrow); (**C**) sagittal T2-weighted MRI through the brain at the level of the ventricles showing multiple well-defined foci of increased signal intensity seen adjacent to the anterior aspect of periventricular white matter; (**D**) coronal T2-weighted MRI through the spinal cord showing well-defined foci of increased signal intensity (Red Arrow).

A 40-year-old female patient reported to the King Abdulaziz University, Faculty of Dentistry/University Dental Hospital (KAU-FD/UDH), with the relapsing–remitting type of MS diagnosed in the year 2020 (Figure 2A–D). She was complaining of bleeding gums with a burning sensation, halitosis, and inability to brush due to pain.

She had been followed-up for 2 years in a private clinic, and later, due to high cost of treatment and lack of insurance coverage, she was shifted to the National Guard Hospital (NGH). She was under medication, including ocrelizumab 600 mg IV every 6 months. Medically, she was fit and experiencing no other systemic disorders other than MS. However, she had a history of gestational DM. The outbreaks started 9 years ago but remained undiagnosed until the recent episode, which happened in 2020 after receiving the COVID-19 vaccination. It manifested as unilateral numbness and heaviness in the right side of the body. The patient was prescribed with ocrelizumab 600 mg IV every 6 months along with oral prednisolone 20 mg/day. She was also taking other medications, including analgesics, pantoprazol, and levocetirizine, for headaches, stomachaches, and allergies, respectively, which accompanied with the IV dosages.

Due to her medical condition, she reported a compromised social life, being at home for most the time, and missed prominent events concerning her family and friends. On questioning, she also revealed her smoking habit of 4–5 cigarettes/day.

**Figure 3:**
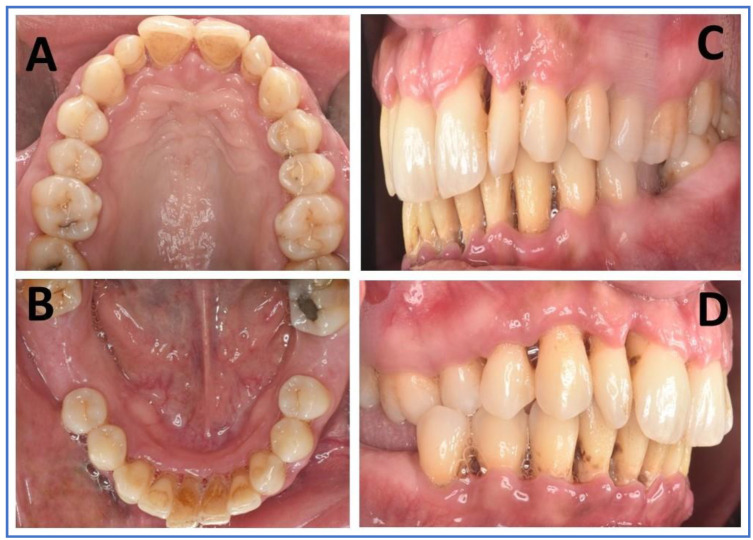
Intraoral images (**A**–**D**): (**A**) maxillary arch in palatal view with deposits in palatal aspect of anterior teeth, as well as composite restorations and dental caries in posteriors; (**B**) mandibular arch in lingual view showing deposits on the lingual aspect of anterior teeth, and the missing first molars of either side; (**C**,**D**) right and left lateral aspects showing deposits and gingival inflammation of marginal and interdental papillae, along with gingival recession.

**Figure 4:**
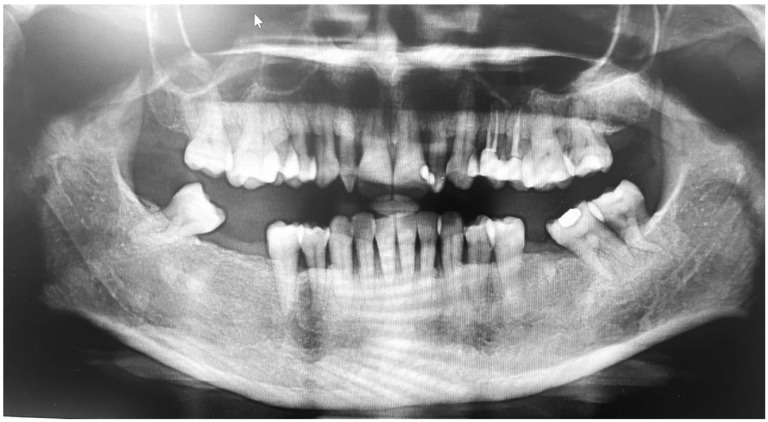
Orthopantomogram showing generalized horizontal bone loss, multiple missing teeth, and restorations.

Extraoral examinations were within normal limits, focusing on facial symmetry, temporomandibular joint (TMJ), and potential trigger points of trigeminal neuralgia (TN). Upon intraoral examination, the patient had localized gingival inflammation in the upper and lower anterior areas, with punched-out papillae similar to necrotizing uncreative gingivitis (NUG). There was gingival recession of 2–7 mm, along with visible plaque and calculus in the same area and associated halitosis (Figure 3A–D). However, the pocket depth was not recorded due to severe pain upon touch, with a score of 9 out of 10 measured on the visual analog scale (VAS). Active dental caries at #16, 17 and previous restorations at teeth #24, 25, 37 were also observed (Figure 4).

At the end of the initial visit, chlorhexidine mouthwash (0.12% bid) was prescribed, along with oral hygiene instructions (OHIs) that include a modified Stillman brushing technique to avoid touching the gingiva. Consultations were conducted with the department of oral medicine and department of prosthetic dentistry, and with her physician, regarding the medications, planned dental treatment, and any medical considerations or drug interactions.

During the subsequent visit after 1 week, the aim was to further reduce the bacterial load. Accordingly, full-mouth ultrasonic scaling with light supra-gingival scaling only at the anterior teeth was performed under topical anesthesia with OHI re-enforcement, as she continued to complain of pain when the gingiva were touched. On visual analogue scale, the patient had a scoring of 4 out of 10. The Ainmo and Bay bleeding index (BI) was 76%, with mobility of grade III at #31, #32, #41, and #42, grade II at #12, #22, and grade 1 at #11, #21, #33, and #43, with probing pocket depth (PPD) average of ≥5 mm in more than 30% of the sites involved. Furthermore, the clinical attachment loss was ≥5 mm #31, #32, #41, #42, #12, #13, #21, and #22. With the above description, the case was diagnosed as stage III and grade B periodontitis.

At the third visit, great improvement was seen regarding gingival inflammation and bleeding. Since the pain was reduced (1 out of 10 VAS), sub-gingival scaling and root planning was carried out, followed by OHI re-enforcement. Owing to the improved gingival condition, a modified Bass technique of brushing with interdental brushes and water floss was advised. Additionally, a combination antibiotics (augmentin 625 mg bid plus metronidazole 500 mg bid) similar to the protocol of treating aggressive periodontitis and necrotizing ulcerative gingivitis cases was prescribed [72].

On the first follow-up visit after 1 month, the bleeding index was reduced to 15%, with a normal pale pink color of the gingiva and better consistency, clinical attachment loss (CAL) #11, #21, #13, #23, #33, #43 ranging between (2–4 mm), mobility of grade 1 at #11, #21, and PD from 6 mm to 3 mm with reduced but stable periodontium. Initially, restorations were conducted in #16, #17, followed by open flap debridement in relation to #25, #26 and #37, #38. Later, fiber splinting was conducted in the lower anterior (33–43). Lastly, implants were placed in #36, #46, and #47.

## 7. Conclusions

Multiple sclerosis is a demyelinating disorder with an unknown etiology. However, there are a few factors, such as vitamin D deficiency, EBV and HHV-6 infections, that are known to be associated with it. It is considered as a form of T-cell-mediated destruction of oligodendrocytes and associated myelin sheaths of neurons. The clinical manifestations are varied depending on the involved area of the CNS. The disease is more commonly seen in young females, with optic neuritis being the initial symptom. Other manifestations include muscle spasm, ataxia, and peripheral neurological disturbances. Diagnosis is achieved through MRI examination, in addition to lumbar puncture and evoked potential study of the nerves. The treatment includes high doses of corticosteroids, plasmapheresis, and biologics.

Oral manifestations include burning sensation, numbness, TN, and orofacial pain. Also seen are high prevalences of periodontal diseases, dental caries, reduced saliva, and halitosis. As a dentist, if any abnormalities are encountered during the clinical examination or while examining the cranial nerve in the risk group, a consultation with the neurologist is mandatory. Trigeminal neuralgia may be an initial manifestation in 5% of patients, and glossopharyngeal neuralgia was reported in 0.5% of patients. Dentists not only bear responsibility to deliver treatment, but also should be cautious about the possible drug interactions, and consider patients on long-term corticosteroid therapy undergoing dental treatment. Dentists should also identify and treat the side effects of drug therapy given for the treatment of MS. More studies in relation to MS and dental management is required to raise the awareness among the dental community about these rare, but rising, cases in the community.

## Figures and Tables

**Table 1:** Search strategy used for different databases.

Database	Search Strategy
PubMed	“Multiple Sclerosis” [Mesh] OR “Demyelinating Autoimmune Diseases, CNS” [Mesh] OR “relapsing–remitting” [tw] AND “Oral Manifestations” [Mesh] OR “Oral Health” [Mesh] OR Mouth [tw] OR “oral health care” [tw] OR “oral mucous irritation” [tw]
SCOPUS	(TITLE-ABS-KEY (multiple AND sclerosis) AND TITLE-ABS-KEY (oral AND mucous AND irritation) OR TITLE-ABS-KEY (trismus) OR TITLE-ABS-KEY (xerostomia) OR TITLE-ABS-KEY (gingivitis))
Cochrane	multiple sclerosis in Title Abstract Keyword AND trismus in Title Abstract Keyword OR oral mucosal irritation in Title Abstract Keyword OR gingivitis in Title Abstract Keyword OR xerostomia in Title Abstract Keyword—(Word variations have been searched)

**Table 2:** Studies reporting the orofacial manifestations of MS (Multiple Sclerosis).

Author	Study Design	Sample Size and Intervention	Outcome and Key Findings of the Study
Covello F et al. [39]	Cross-sectional study	A total of 101 patients with MS were interviewed with three different questionnaires to evaluate the different aspects of MS, including status of oral health, level of functional disability, dysphagia, and quality of life.	The responses from three questionnaires, namely, oral hygiene test, dysphagia in MS (DYMUS), and oral health impact on quality of life (IOHIP), showed non-significant differences between gender, except for one question—“difficulty in relaxing”. Majority of participants were reported to have paraesthesia of soft tissues, such as lips and gums. Dysphagia and dysarthria were a common reported symptom among the participants. The quality of life of participants was reported to be compromised due to symptoms, including gingivitis, dry mouth, numbness, and difficulty swallowing.
Symons AL et al. [59]	Cross-sectional study	A total of 22 patients were interviewed regarding their past medical and dental records. Both extra-oral and intra-oral clinical examinations were carried out.	Based on dental caries and periodontal indices, the MS patients were not reported to have any significantly higher risk. However, the symptoms of TN and TMJ disorders were found to be more prevalent, and need attention and care.
Cruccu G et al. [60]	Multicenter, cross-sectional study	A total of 130 patients of MS were recruited. Based on the diagnosis, they were categorized into three groups—MS with TN (n = 50), MS with sensory disturbance other than TN (n = 30), and control subjects (n = 50). All subjects underwent pain assessment and trigeminal reflex testing. The retrieved MRI scans were incorporated into brainstem models.	The results suggest that the development of TN in predisposed MS patients is probably due to the lesion at intra-pontine trigeminal primary afferents. The ages for the onset of disease and the symptoms of TN were found to be significantly greater in the subjects with TN.
Huggins HA et al. [61]	Cross-sectional study	The biochemical changes in the CSF were labeled and monitored after the removal of amalgam and other dental materials.	With the process of photolabeling of CSF proteins, marked changes were identified, which suggests that these can be potentially used as markers to monitor MS.
Santa Eulalia-Troisfontaines E et al. [62]	Cross-sectional study	A total of 64 MS patients in three different age groups were recruited. Complete oral examination, including various caries and periodontal indices, were measured.	The caries prevalence was found to be comparable with the general population. However, periodontal, especially the gingival, health was compromised, and needs more attention and care.
Sheu JJ et al. [63]	Population-based, case–control study	A total of 316 MS patients, along with 1580 control patients, were recruited in the study. The study attempted to investigate the association between MS and chronic periodontitis.	The statistical analysis revealed that female MS patients have a 1.86 times higher chance of having CP compared to the control counterpart, whereas no such association was established for male MS patients.
Danesh-Sani SA et al. [41]	Cross-sectional study	A total of 500 MS patients were included in the study. Their past medical records, along with sociodemographic data, were gathered. All patients underwent standard neurological examination to assess MS-associated conditions, especially TN, dysphagia, and associated difficulty in speech.	The study found that TMJ disorders, difficulty in speech, and disturbance of vision to be the most commonly reported disorders. It was recommended that the awareness of dental practitioners should be raised regarding the medications routinely prescribed to MS patients, so as to avoid any drug-related interactions.

Note: DYMUS, DYsphagia in MUltiple Sclerosis; IOHIP, Italian version Oral Health Impact Profile; TN, Trigeminal Neuralgia; CSF, Cerebrospinal fluid; CP, Chronic Periodontitis; TMJ, Temporomandibular Joint.

**Table 3:** Summary of drug therapy of MS patients and their adverse side effects [65,66,67,68,69,70,71].

Drug	Adverse Side-Effects	Monitoring	Drug Interactions
Corticosteroids	Adrenocortical deficiency Delayed wound healing Postoperative infections Osteoporosis	Fasting blood sugar Bone density	Aspirin Ciprofloxacin Fluconazole Warfarin
Monoclonal antibody	Oral lichenoid drug-induced eruptions MRONJ	Virus infections Full blood count Liver function test	Tramadol Zidovudine Acetaminophen
Azathioprine	Risk of infections Gastric irritation Hepatotoxicity Nephritis Malignancies on long-term use	Thiopurine S-methyltransferase (TPMT)	Aspirin Co-trimoxazole Warfarin
Methotrexate	Allergic reactions Hepatotoxicity Nephrotoxicity	Complete blood count, serum creatinine, transaminases	Amoxicillin Aspirin Omeprazole
Cyclophosphamide	Infections Malignancies Infertility	Fasting blood sugar Liver function test Renal function test	Lidocaine Metronidazole Zidovudine

Note: MRONJ, medication-related osteonecrosis of the jaw.

## Data Availability

Data will be made available on reasonable request from the corresponding author.

## Abbreviations

MS	Multiple Sclerosis
TN	Trigeminal Neuralgia
RRMS	Relapsing–Remitting Multiple Sclerosis
SPMS	Secondary Progressive Multiple Sclerosis
PPMS	Primary Progressive Multiple Sclerosis
PRMS	Progressive–Relapsing Multiple Sclerosis
EBV	Epstein–Barr Virus
HHV-6	Human Herpesvirus 6
HSR	Hypersensitivity Reaction
BBB	Blood–Brain Barrier
IL-1	Interleukin-1
IL-6	Interleukin-6
IL-10	Interleukin-10
INF-γ	Interferon gamma
TNF-α	Tumor Necrosis Factor Alpha
TGF-β	Transforming Growth Factor Beta
CNS	Central Nervous System
WBCs	White Blood Cells
CSF	Cerebrospinal Fluid
MRI	Magnetic Resonance Imaging
VEP	Visual Evoked Potential
QoL	Quality of Life
